# Recent Progress in the Integration of CO_2_ Capture and Utilization

**DOI:** 10.3390/molecules28114500

**Published:** 2023-06-01

**Authors:** Huanghao Ning, Yongdan Li, Cuijuan Zhang

**Affiliations:** 1 Tianjin Key Laboratory of Applied Catalysis Science and Technology, State Key Laboratory of Chemical Engineering, School of Chemical Engineering and Technology, Tianjin University, Tianjin 300072, China; hhning@tju.edu.cn (H.N.); yongdan.li@aalto.fi (Y.L.); 2 Collaborative Innovation Center of Chemical Science and Engineering (Tianjin), Tianjin 300072, China; 3 Department of Chemical and Metallurgical Engineering, School of Chemical Engineering, Aalto University, Kemistintie 1, P.O. Box 16100, FI-00076 Espoo, Finland

**Keywords:** carbon neutrality, CO_2_ capture, CO_2_ conversion, integration

## Abstract

CO_2_ emission is deemed to be mainly responsible for global warming. To reduce CO_2_ emissions into the atmosphere and to use it as a carbon source, CO_2_ capture and its conversion into valuable chemicals is greatly desirable. To reduce the transportation cost, the integration of the capture and utilization processes is a feasible option. Here, the recent progress in the integration of CO_2_ capture and conversion is reviewed. The absorption, adsorption, and electrochemical separation capture processes integrated with several utilization processes, such as CO_2_ hydrogenation, reverse water–gas shift reaction, or dry methane reforming, is discussed in detail. The integration of capture and conversion over dual functional materials is also discussed. This review is aimed to encourage more efforts devoted to the integration of CO_2_ capture and utilization, and thus contribute to carbon neutrality around the world.

## 1. Introduction

Carbon dioxide capture, utilization, and storage (CCUS) are increasingly gaining global attention. The challenge is to meet the energy demand while balancing CO_2_ emissions. Several solutions have been proposed to reduce the CO_2_ emission, namely, increasing the utilization of eco-friendly energy sources, such as wind and solar energy, to improve the energy efficiency. However, the advancement of such technologies is currently still limited and can be an optimal option in the long-term. At present times, the CCUS technology is more effective and can be a short-term alternative.

CO_2_ can be captured using various technologies, such as amine absorption, porous materials adsorption and membrane separation. Since CO_2_ itself is a carbon source, it can be converted into valuable chemicals via dry methane reforming (DMR), CO_2_ hydrogenation, reverse water–gas shift reaction, etc. There are excellent reviews on both the technologies [1,2,3]. However, the traditional CO_2_ capture and utilization processes are separated, which inevitably increases the transportation cost. To reduce or even eliminate such cost, the integration of CO_2_ capture and utilization is greatly desirable. This review focuses on the integration of CO_2_ capture and utilization. The relevant processes and the materials involved will be discussed.

## 2. Integration of CO_2_ Capture and Utilization

Integrated CO_2_ capture and utilization aims to capture CO_2_ from gas streams and other emission sources and convert it into valuable chemicals or energy sources. The key is to find the match between the CO_2_ separation process and the CO_2_ utilization process, including the temperature and pressure, etc.

### 2.1. Integration of CO_2_ Absorption and Conversion

CO_2_ can be captured via physical or chemical absorption depending on the interaction between CO_2_ and the absorbent. The former mainly utilizes the solubility of each gas component in the solvent whereas the latter involves chemical reactions between CO_2_ and the absorbent. Chemical absorption is mostly employed with organic amine, hot potash, and liquid ammonia solvents considering its easy operation and mild working conditions [4]. Accordingly, it can be integrated with CO_2_ hydrogenation for the production of formic acid or methanol.

**Figure 1:**
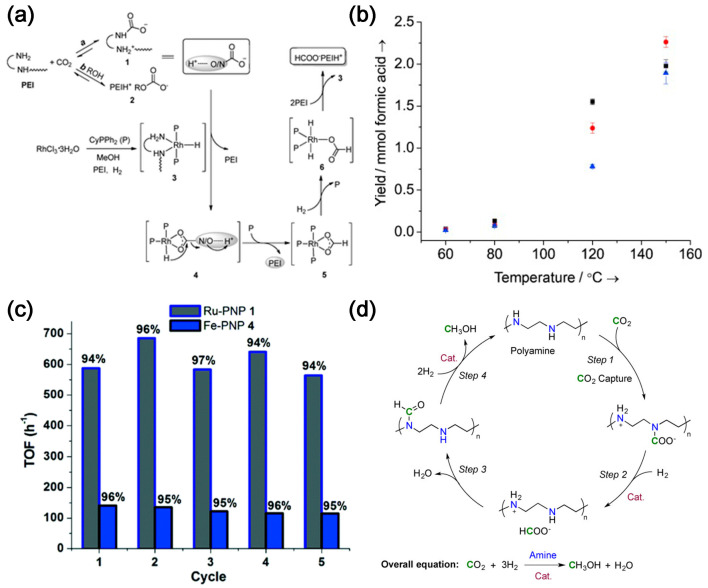
(**a**) Proposed pathways of carbon capture and subsequent hydrogenation of the captured CO_2_ [12]; (**b**) Formic acid yields in the hydrogenation of CO_2_ catalyzed by the PEI–PN/Ir materials as a function of temperature and MW of PEI for PEI_600_–PN/Ir (▪), PEI_1800_–PN/Ir (•), and PEI_25 000_–PN/Ir (▴) [13]. (**c**) Multiple recycling of the catalyst in biphasic reaction mixture. Yield (%) of formate is relative to the amount of CO_2_ captured. Ru–PNP 1: Cat 1 = 10 μmol, T = 55 °C, H_2_ = 50 bar, 17.2 mmol diazabicyclo[2.2.2]octane (DABCO) + 3 mL H_2_O (CO_2_ captured each cycle = 15 mmol), 3 mL additional H_2_O–4 mL 2-Methyltetrahydrofuran (2–MeTHF) added for hydrogenation study. Fe–PNP 4: Cat 4 = 20 μmol, T = 55 °C, H_2_ = 50 bar, 17.2 mmol DABCO + 3 mL H_2_O (CO_2_ captured each cycle = 15 mmol), 3 mL additional H_2_O–4 mL 2–MeTHF added for hydrogenation study [14]. (**d**) Proposed reaction sequence for CO_2_ capture and in situ hydrogenation to CH_3_OH using a polyamine [5].

Amine-based CO_2_ capture and conversion integrated reactors consist of a series of amine sorbents and metal ions that form a pincer complex, such as pincerpentaethylenehexamine (PEHA), pyrrolizidine, N,N,N′,N″,N″-pentamethyldiethylenetriamine (PMDTA) and polyethyleneimine (PEI). These are coupled with metal pincer-based homogeneous catalysts [5,6,7,8]. Due to the presence of multiple amine sites [9], PEI can be used as a superior CO_2_ absorber [10], which can absorb both high and low concentrations of CO_2_ [11]. Li et al. [12] used PEI/RhCl_3_·3H_2_O/CyPPh_2_ to capture CO_2_ and convert it in situ (Figure 1a). PEI absorbs CO_2_ from air in an ethylene glycol solution containing PEI and converts it into amino formate esters and alkyl carbonate esters. CO_2_ is hydrogenated in situ to form formate salts (TON = 260) in the presence of RhCl_3_-3H_2_O/CyPPh_2_, demonstrating the first in situ CO_2_ capture and conversion to formate. Multifunctional materials can be synthesized by modifying the PEI backbone with iminophosphine ligand functionality and subsequently metallizing it with Ir precursors. About 65% of the available primary amines on PEI can be modified to form iminophosphine/Ir (PN/Ir) for balanced CO_2_ capture and conversion, resulting in higher formic acid yields. PEI with relatively lower molecular weight has better CO_2_ capture ability and catalytic activity (Figure 1b) [13]. Kothandaraman et al. [14] reported, for the first time, a green and simplified method for in situ conversion of captured CO_2_ to formate in aqueous media in the presence of Ru- and Fe-based pincer complexes without excess alkali, with yields of up to 95% of the formate.

In addition to formate, the captured CO_2_ can be converted into methanol. In 2015, Rezayee et al. [15] prepared methanol by tandem CO_2_ capture and in situ conversion of dimethylamine with homogeneous ruthenium complexes under basic conditions. Dimethylamine can react with CO_2_ and inhibit the formation of formic acid. The conversion of CO_2_ is >95% (Figure 1c). Moreover, Kothandaraman et al. [5] first proposed and demonstrated a system for capturing CO_2_ from air (~400 ppm) and converting it in situ (Figure 1d), which consisted of PEHA and Ru–PNP complex, with a methanol yield of 79%. Integrated systems for CO_2_ capture and conversion into methanol are still uncommon. The major obstacle is the harsh reaction conditions to produce methanol, which involves a high-pressure gas-phase catalyst reactor at relatively low temperatures of 200–300 °C and high pressures of 50–100 atm.

The amine-based materials offer the potential to capture and convert CO_2_ under mild conditions. Due to the relatively high CO_2_ capture capacity of PEI, it can directly capture CO_2_ from the air, which also removes the limitation of constructing CO_2_ capture equipment. However, the high selectivity of some materials to carbon dioxide poses a challenge for the regeneration of amines. Furthermore, the toxicity and corrosiveness of amine solvents limit their industrial application. To drive future research, it is essential to explore systems that are highly efficient and recyclable, enabling CCU to establish a reliable foundation for industrial applications.

### 2.2. CO_2_ Adsorption and Conversion Integration

Similar to absorption, adsorption separation can also be divided into chemisorption and physisorption according to the interaction between CO_2_ and the adsorbate, with the former forming covalent bonds whilst the latter forms bond with electrostatic attraction and van der Waals forces. Here, we mainly focus on the physisorption and the subsequent utilization of CO_2_.

Porous organic polymers (POPs) are a series of new two- or three-dimensional networked polymeric materials formed by covalent bonding of organic small molecule substrates through specific chemical reactions, usually with microporous, mesoporous or multistage pore structures. They have promising applications in separation, sensor and catalysis [16]. The ionic porous organic polymers (IPOPs) are generally classified as porous organic materials, whose backbone typically includes anions or cations. They can be divided into IPOPs with cationic moieties, IPOPs with anionic moieties, and IPOPs with zwitterionic moieties. Common cationic moieties include imidazolium, pyridinium, viologen, and quaternary phosphonium, whereas more common anionic moieties include tetrakis(phenyl)borate and tris(catecholate) phosphate. The inclusion of these ionic moieties in porous materials can enhance their CO_2_ capture capacity and catalyze the in situ CO_2_ conversion.

**Figure 2:**
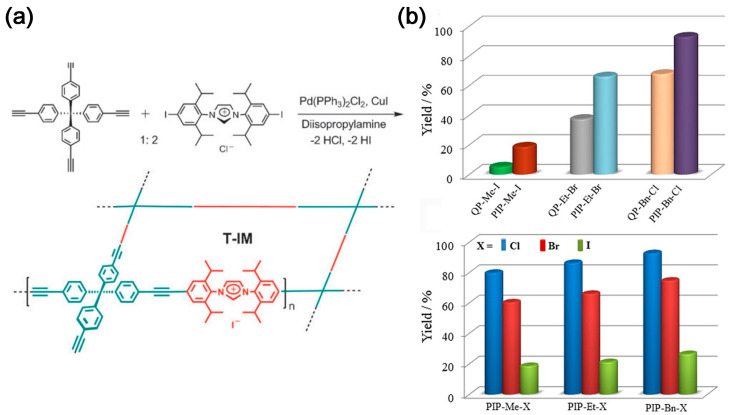
(**a**) Preparation of porous organic networks bearing imidazolium salts [17]; (**b**) Yields of chloropropene carbonate from the cycloaddition of epichlorohydrin and CO_2_ catalyzed by PIPs with corresponding QPs and PIP-Me-X, PIP-Et-X, and PIP-Bn-X (X = Cl, Br, and I). Reaction conditions: epichlorohydrin (1.0 g, 10.9 mmol), catalyst (0.05 mmol, based upon the quaternary phosphonium salt), 323 K, CO_2_ (ambient pressure), and 24 h [19].

In 2011, tetrakis(4-ethynylphenyl)methane and diiodoimidazolium salts were used to prepare tubular microporous organic via Sonogashira coupling reaction networks bearing imidazolium salts (T-IM) (Figure 2a). The material, with a microporous structure of specific surface area (620 m^2^ g^−1^), shows good catalytic activity towards the conversion of CO_2_ to cyclic carbonates [17]. Wang et al. [18] utilized the Friedel–Crafts reaction to synthesize imidazole-based IPOPs. The specific surface area can reach up to 926 m^2^ g^−1^; however, its CO_2_ capture capacity is only 14.2 wt%. Nevertheless, the polymer exhibits good stability and repeatability. Sun et al. [19] demonstrated, for the first time, the capture and in situ conversion of CO_2_ into cyclic carbonates under relatively mild room temperature conditions using a metal-free solvent followed by a heterogeneous catalytic system. The effect of halogen anions (Cl, Br, and I) and quaternary phosphonium cations on the catalytic activity was investigated. The catalytic activity follows the order Cl^−^ > Br^−^ > I^−^ (Figure 2b), because the rate-controlling step of the reaction is ring opening by anion attack on the epoxide [20].

In addition to IPOP, the porphyrin-based organic polymer (POP-TPP) can also be used. Xiao et al. [21] synthesized a hierarchically porous organic polymer (POP-TPP) by polymerizing vinyl-functionalized tetraphenylporphyrin monomer, and then metalated it with different metals (Co^3+^, Zn^2+^, and Mg^2+^). The resulting heterogeneous catalysts have rich active sites and exhibit higher activity than the homogenous Co/TPP catalyst at relatively low CO_2_ concentrations, primarily due to the favorable enrichment of CO_2_ in the porous structure (micropores and nanochannels) of Co/POP-TPP. The TOF of the catalysts decreases in the following order: Co/POP-TPP (436 h^−1^) > Zn/POP-TPP (326 h^−1^) > Mg/POP-TPP (171 h^−1^) (Table 1). Later, a series of high surface area hollow tubular metal (Al, Co, Fe, and Mn) porphyrin-based hypercrosslinked polymers (HCP) were synthesized via Friedel–Crafts alkylation reactions. Al-HCP can catalyze the formation of propylene carbonate with a selectivity of approximately 99% after only 1 h with 2.0 mol% TBAB catalyst [22].

**Figure 3:**
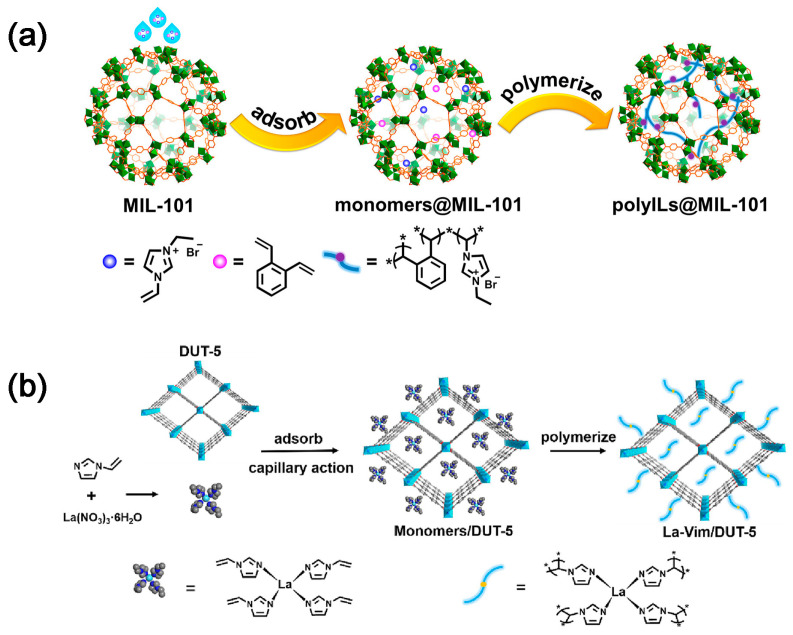
Schematic illustration of the preparation of (**a**) polyILs@MIL–101 [24] and (**b**) La–Vim/DUT–5 [27]. The “*” represents the monomer in the polymer.

Metal–organic frameworks (MOFs) are porous crystalline materials formed by coordination between metal ions or metal clusters and organic ligands, which are characterized by high porosity, high surface area, tunable pore size and geometric configuration, and functionalizable pore surface [23]. Consequently, they can be employed for CO_2_ capture and utilization. The imidazolium-based poly(ionic liquid)s (denoted as polyILs) and ortho-divinylbenzene were used as cross-linking agents to polymerize in the pores of MIL-101 (Figure 3a), resulting in polyILs@MIL-101 materials with dual functions of CO_2_ capture and conversion [24]. For MOFs materials, ordered nanochannels can effectively promote the enrichment of CO_2_ at the active sites and accelerate the CO_2_ conversion; Lewis basic sites (LBSs) can supply electrons to activate CO_2_ [25,26]. In order to restore the real CO_2_ capture process, a CO_2_ capture and in situ conversion system was designed by simulating the flue gas feed, where the lanthanide (III) complex of 1-vinylimidazole (Vim) was immobilized with DUT-5 by a combination of ligand and in situ polymerization (Figure 3b). This work opens up a general pathway for future research and validates the effectiveness of MOFs for practical applications in CO_2_ capture and conversion [27].

Although the integrated materials have demonstrated good CO_2_ capture and enrichment capabilities, their performance under the real industrial exhaust emissions that contain acidic gases, such as SO_x_, NO_x_, and steam, remain to be explored.

### 2.3. CO_2_ Electrochemical Membrane Separation and Conversion Integration

Electrochemical membrane separation is a technology that achieves gas separation through electrochemical reaction. The membrane can be mixed oxide ion-carbonate conductor (MOCC), such as Y_0.16_Zr_0.84_O_2-δ_-molten carbonate (YSZ-MC), Ce_0.8_Sm_0.2_O_1.9_-MC (SDC-MC), Ce_0.9_Gd_0.2_O_1.9_-MC (GDC-MC), and Bi_1.5_Y_0.3_Sm_0.2_O_3-δ_-MC (BYS-MC); mixed electron-carbonate conductor (MECC), such as stainless steel-MC (SS-MC), Ag-MC, and Ni-MC; or mixed oxide ion-electron-carbonate conductor (MOECC), such as La_0.6_Sr_0.4_Co_0.8_Fe_0.2_O_3-δ_-MC (LSCF-MC), La_0.5_Sr_0.5_Fe_0.8_Cu_0.2_O_3-δ_-MC (LSFCu-MC) and NiO-SDC-MC based on the charge carriers [2]. Generally, the carbonates (Li-Na-K-based) show high electrical conductivity and low viscosity only at relatively high temperature, e.g., >600 °C. Accordingly, it can be well integrated with the high temperature CO_2_ utilization processes, such as DMR and reverse water–gas shift (RWGS) (Table 2).

**Figure 4:**
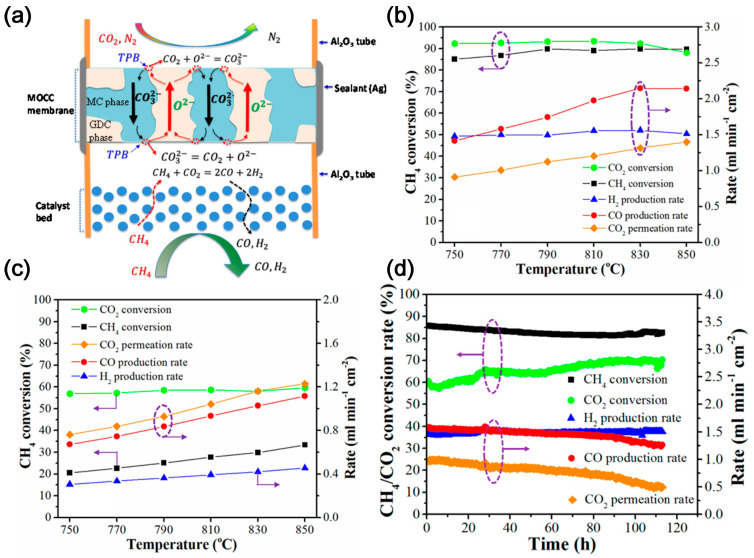
(**a**) Schematic illustration of a MOCC membrane reactor with a catalyst bed for electrochemical CO_2_ capture and catalytic DMR. TPB: triple phase boundary [31]. (**b**) Effect of temperature on the DMR performance of a GDC–MC membrane reactor with an NMP catalyst [31]. (**c**) Effect of temperature on the DMR performance of a GDC–MC membrane reactor with an NMP catalyst and an LNF catalyst [31]. (**d**) Stability of DOMR performance of the Ag–MC MECC membrane reactor at 800 °C with NMP catalyst. Feed gas: 75 mL min^−1^ N_2_, 15 mL min^−1^ CO_2_, and 10 mL min^−1^ O_2_; sweep gas: 0.94 mL min^−1^ CH_4_ and 50 mL min^−1^ Ar [32]. The dotted circles and arrows in the figure represent the axes corresponding to the curve.

Lin and co-workers first modeled the high temperature tube shell membrane reactor for separation and utilization of CO_2_ from the flue gas and for simultaneous production of syngas using DMR. The membrane reactor is highly efficient for CCUS. The CH_4_ conversion of 48.1% and an average CO_2_ permeation flux of 1.52 mL cm^−2^ min^−1^ can be obtained at 800 °C with a 75 µm thick membrane and CH_4_ space velocity of 3265 h^−1^ [28]. Later, Anderson et al. [29] experimentally studied the integration of CO_2_ separation and DMR with LSCF-MC membrane and Ni/γ-alumina catalyst. The CO_2_ permeation rate above 750 °C matches the reaction rate of DMR catalyst, and the order of syngas production activity is blank system < LSCF combustion catalyst < Ni/γ-alumina reforming catalyst. The conversion of CO_2_ and CH_4_ is 88.5 and 8.1%, respectively, generating a syngas H_2_:CO ratio of about 1 [30]. To improve the performance, Zhang et al. [31] employed MOCC membrane (GDC-MC) (Figure 4a), and NMP (Ni-MgO-1 wt% Pt) and LNF (LaNi_0.6_Fe_0.4_O_3-δ_) catalysts. The yield of H_2_ and CO and the conversion of CH_4_ in the reactor with NMP catalyst is higher than that with LNF catalyst (Figure 4b,c), while the reactor with LNF catalyst shows better anti-coking performance and no significant degradation within 200 h. In addition, Zhang et al. [32] investigated the MECC membrane reactor (Ag-MC) coupled with dry-oxy methane reforming (DOMR) over an NMP catalyst. The CH_4_ conversion is >82% and is stable over 115 h at 800 °C (Figure 4d). Similar reactor models can be applied to integrate CO_2_ separation and oxidative dehydrogenation of ethane (ODHE) to prepare ethylene [33]. CO_2_ can react with H_2_ to make the reaction of ethane dehydrogenation forward, which can increase the selectivity of O_2_-ODHE to produce ethylene [33].

The coupling of a dual-phase membrane reactor with a RWGS reaction was first reported by Chen et al. [34] to capture CO_2_ and produce CO. Under a sweep gas condition of 1% H_2_/He, the CO selectivity over LaNiO_3_ (LNO) catalyst is >96% at 550–750 °C. Additionally, La_0.9_Ce_0.1_NiO_3-δ_ (LCNO) catalyst displays almost 100% CO selectivity and good thermal stability. The introduction of H_2_ during the purge test enhances the permeation of CO_2._ H_2_O in dual-phase membrane systems can reduce the activation energy, possibly due to the conduction of hydroxide ions through the membrane [35].

**Figure 5:**
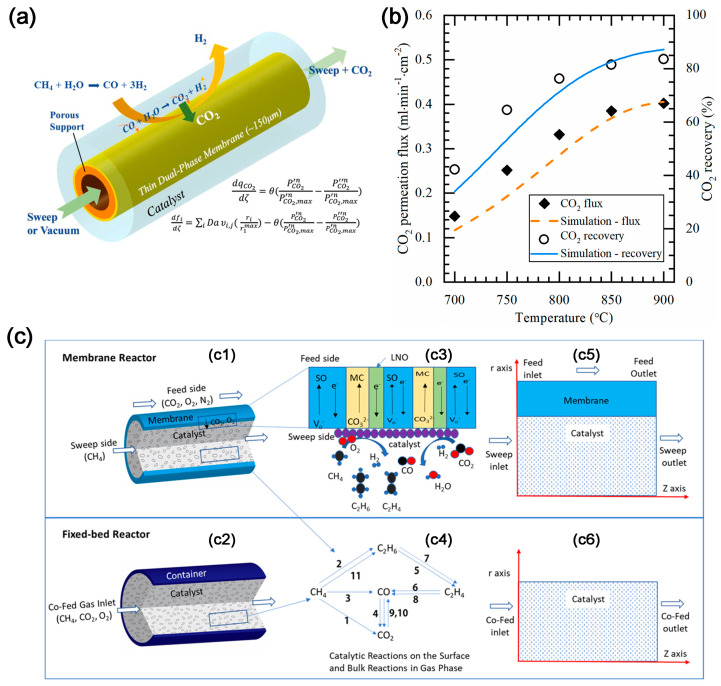
(**a**) Schematic illustration of membrane reactor [37]. (**b**) Comparison of CO_2_ flux and recovery of the membrane reactor between experimental results and modeling results. Conditions: sweep: He = 200 mL min^−1^; feed: CH_4_ = 5 mL min^−1^; S/C = 3; both sides of the membrane were at 1 atm [37]. (**c**) Schematic illustration of (**c1**) Membrane reactor; (**c2**) Co-fed fixed bed reactor; (**c3**) Charge species transport and surface reactions in the membrane reactor; (**c4**) Reaction pathway diagram. 2D axial symmetric computational domain of (**c5**) Membrane reactor; (**c6**) Co-fed fixed bed reactor [38].

The dual-phase membrane reactor can also increase the H_2_ yield by removing CO_2_. The concept of CO_2_ removal was first applied to the steam reforming of methane reaction (SMR) to produce high concentration of H_2_ by Wu et al. [36]. The asymmetric BYS-SDC-MC membrane reactor can convert methane into hydrogen via SMR reaction while simultaneously removing CO_2_ to achieve a high concentration of hydrogen, with 84% CO_2_ recovery (Figure 5a). The results of the mathematical model (Figure 5b) demonstrate that CO_2_ recovery from SMR in the CO_2_ permeated membrane reactor exceeds 99% and a pure H_2_ gas stream without CO gas can be achieved [37].

The dual-phase membrane reactors can also be utilized for the oxidative coupling of methane (OCM) reaction. The high oxygen partial pressure in conventional OCM reactors often results in low C_2_ selectivity. Li et al. [38] combined CO_2_/O_2_ transport membrane (CTM) and OCM reaction to build a new membrane reactor model (Figure 5c), which shows higher conversion of CH_4_ than the traditional fixed-bed reactor model and better coking resistance. Based on this, Zhang et al. [39] developed a membrane reactor combining SDC-NiO-MC and 2%Mn–5%Na_2_WO_4_/SiO_2_ catalyst. The co-captured CO_2_/O_2_ mixture converts CH_4_ into C_2_H_6_ over the 2%Mn–5%Na_2_WO_4_/SiO_2_ catalyst, followed by thermal cracking into C_2_H_4_ and H_2_. In the presence of CO_2_, the O_2_ partial pressure is reduced, thereby reducing the possibility of re-oxidation of C_2_ products, which leads to a higher C_2_ selectivity.

**Table 2:** Summary of electrochemical membrane materials integrated reactors.

Membrane	Catalyst	Reaction	Ref.
La_0.6_Sr_0.4_Co_0.8_Fe_0.2_O_3-δ_ Li-Na-K	10 wt%Ni-/γ-Al_2_O_3_	DMR	[29]
Ce_0.8_Gd_0.2_O_1.9_ Li-Na	Ni-MgO-1 wt% Pt LaNi_0.6_Fe_0.4_O_3-δ_	DMR	[31]
Ag Li-Na	Ni-MgO-1 wt% Pt	DOMR	[32]
NiO-SDC Li-Na	Ni-MgO-1 wt% Pt	DOMR	[40]
Ce_0.8_Gd_0.2_O_1.9_ Li-Na	5 wt% Cr_2_O_3_- ZSM-5	Ethane-to-Ethylene	[33]
Ce_0.9_Pr_0.1_O_2-δ_-Pr_0.6_Sr_0.4_Fe_0.5_Co_0.5_O_3-δ_ Li-Na-K	10 wt%Ni-/γ-Al_2_O_3_	DOMR	[41]
LNO/SDC Li-Na	LNO/LCNO	RWGS	[34]
BYS-SDC Li-Na-K	Ni-based catalyst (HiFUEL R110)	SMR	[36]
γ-LiAlO_2_-Ag Li-Na-K	γ-LiAlO_2_-Ag	Syngas production	[42]
NiO-SDC Li-Na	2%Mn-5%Na_2_WO_4_/SiO_2_	OCM	[39]

Note: Li-Na-K = Li_2_CO_3_: Na_2_CO_3_:K_2_CO_3_ with ratio of 42.5:32.5:25 mol%; Li-Na = Li_2_CO_3_: Na_2_CO_3_ with ratio of 52:48 mol%.

### 2.4. CO_2_ Capture and Conversion over Dual-Function Materials (DFMs)

Inorganic metal dual-functional materials (DFMs) combine the capture and conversion of CO_2_ into organic compounds. DFMs consist of the CO_2_ absorption component and the catalytic CO_2_ conversion component. The former is usually composed of alkali metal oxides or carbonates whilst the latter comprises metal-based catalysts, such as Ru, Rh, and Ni for DMR, dry ethane reforming (DER), RWGS reaction, and CO_2_ methanation [43,44,45]. Those processes can be well integrated with CO_2_ absorption considering their similar operating temperatures.

**Figure 6:**
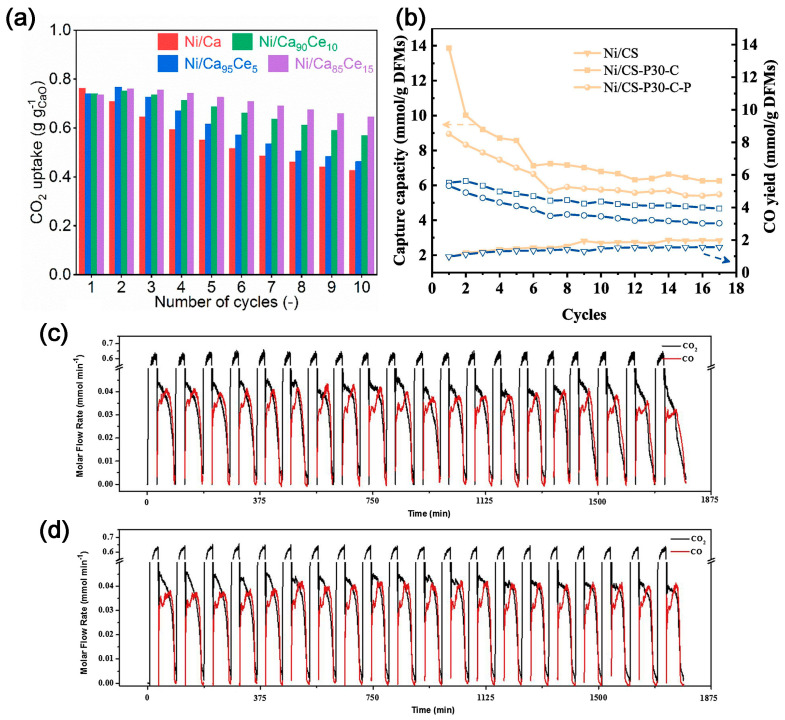
(**a**) Comparison of cyclic CO_2_ capture-release stability of the as-prepared bifunctional materials at 650 °C [48]. (**b**) CO_2_ sorption capacities and CO productivities of Ni/CS, Ni/CS–P30–C and Ni/CS–P30–C–P at 650 °C within 17 cycles [49] (The symbols indicate different preparation methods. The yellow and blue lines represent CO_2_ sorption capacities and CO productivities, respectively). Cyclic CO_2_ capture and conversion reactions of (**c**) Ca_1_Ni_0.1_; and (**d**) Ca_1_Ni_0.1_Ce_0.033_ [50].

In 2018, Kim et al. [46] proposed and demonstrated the feasibility of using DFM to capture CO_2_ and convert it in situ with renewable CaO as an absorber of CO_2_ and Ni/MgO-Al_2_O_3_ as a catalyst for DMR. The concentration of CO_2_ in the exhaust gas is less than 0.08%, and the ratio of H_2_ to CO is 1.06. Tian et al. [47] synthesized CaO-Ni DFM using a sol–gel method. The ratio of H_2_ to CO is 1.1, which is similar to Kim’s results [46]. Methane decomposition can occur simultaneously during the reaction. The deposited carbon facilitates the reaction of CO_2_ with CaO-Ni; the CH_4_ conversion rate can reach 86%. Compared to other processes for DMR, the in situ consumption of CO_2_ on the catalyst surface promotes a positive shift of equilibrium in favor of CaCO_3_ decomposition, which allows the otherwise energy-intensive calcium cycling process to proceed at lower temperatures, thus further alleviating the deactivation problem during calcination. The energy consumed is 22% lower than the conventional consumption. In order to improve the activity and stability of Ni-CaO in the DMR reaction [48], CeO_2_ was added to the support, with a Ca:Ce molar ratio of 85:15. The resultant catalyst demonstrates good stability and maintains ≈ 80–90% activity over nine cycles at 650 °C, which is over two times better than the material without CeO_2_ modification (Figure 6a). The dispersion of Ni and the reducibility of NiO are improved, enhancing the DMR activity. Due to the high mobility of lattice oxygen in CeO_2_, the CeO_2_ modification can inhibit the accumulation of non-active carbon on Ni.

In addition to DMR, the captured CO_2_ can be also converted into methane over DFM (Table 3). The Farrauto group is a pioneer in the study of CO_2_-Met DFM. They prepared DFM using Ru as the metal catalyst, CaO as the CO_2_ absorbent, and γ-Al_2_O_3_ as a carrier [51]. At 320 °C and 10% CO_2_/N_2_, CO_2_ capture was carried out followed by methane production reaction for 2 h with 4% H_2_/N_2_. The 5% Ru-10% CaO/γ-Al_2_O_3_ exhibits the highest activity. In the CO_2_ capture cycle test with presence of steam, the purity of obtained methane can reach up to 99%. Duyar et al. [52,53] synthesized Ru- and Rh-based DFM from the nitrates of Ru and Rh, respectively, via impregnation. The Rh-based DFMs show better activity. However, their high cost limits large-scale application. In order to reduce the cost, Bermejo-Lopez et al. studied DFMs using Ni as the catalyst [54,55]. Ni-CaO/γ-Al_2_O_3_ and Ni-Na_2_CO_3_/γ-Al_2_O_3_ with different Ni loadings were synthesized via impregnation. The methane yield of 10% Ni-Na_2_CO_3_/γ-Al_2_O_3_ is 186 μmol CH_4_ g^−1^ at 400 °C and it is 142 μmol CH_4_ g^−1^ at 520 °C for 15% Ni-CaO/γ-Al_2_O_3_. In addition, Al-Mamoori et al. [56] prepared DFMs using Ni-impregnated CaO- and MgO-based double salts as CO_2_ absorber and catalyst, respectively, and γ-Al_2_O_3_ as the carrier. The CO_2_ absorption was first saturated in a 10% CO_2_/N_2_ atmosphere at 650 °C and 1 bar, and then a 5% C_2_H_6_/N_2_ mixture was passed into the reactor to react with CO_2_. The Ni@(K-Ca)/γ-Al_2_O_3_ system exhibits the highest CO_2_ absorption and 100% C_2_H_6_ conversion. Nevertheless, Ni usually requires high reduction temperature and is very sensitive to O_2_ in the feed gas, and its long-term stability remains to be improved for the CO_2_-Met reaction.

DFMs can also convert the captured CO_2_ into valuable chemicals via RWGS reactions and ethane reforming. To investigate the effect of preparation methods on the performance of DFMs, Wang et al. [49] synthesized DFMs using three different methods, namely wet-mixing, acidification/impregnation, and acidification/impregnation combined with citric acid complexation. The Ni/CS-P30-C, prepared via acid pretreatment of carbide slag followed by citric acid complex, exhibits a high CO_2_ sorption capacity (13.28 mmol g^−1^ DFMs) and a great CO productivity (5.12 mmol g^−1^ DFMs) at 650 °C, and also demonstrates better CO_2_ capture and in situ conversion (Figure 6b). Sun et al. [50] investigated the effect of Ce loading onto the Ca-Ni-based DFM on the performance of CO_2_ capture and RWGS. The DFM with Ca:Ni:Ce = 1:0.1:0.033 (molar ratio) exhibits high cycling stability, and 100% CO selectivity (Figure 6c,d). The addition of CeO_2_ effectively prevents the growth and aggregation of NiO and CaO species.

The primary concerns are catalyst deactivation during the reaction, carbon deposition, and matching capture and conversion rates, along with the requirement of evaluating the life cycle and economics of the integrated system. Therefore, further research is necessary in the future on aspects, such as reaction atmosphere, temperature, life cycle, economic analysis, and industrial applications.

## 3. Conclusions, Challenges and Opportunities

In summary, the integration of CO_2_ capture and utilization is reviewed, namely the absorption, adsorption, and electrochemical separation capture processes integrated with several utilization processes. Initially, it is imperative to ensure that the operational conditions of the CO_2_ capture process and the subsequent reaction process are fundamentally congruent, encompassing factors, such as temperature, pressure, pH, and other relevant parameters. Subsequently, the CO_2_ absorption materials can be more effectively combined with the catalyst without impeding their respective performances. The point is to find the match between the operating temperature of both processes. To improve the performance of the integration system, one of the key factors is to achieve the match between the capture rate and the conversion rate. For example, in the MOCC and DMR system, matching the CO_2_ separation rate of the MOCC membrane and the conversion rate over DMR catalyst is crucial. Unfortunately, such studies are very limited. Of course, improving the performance (activity, and stability) of the single process, either the capture or the conversion process, is desirable.

Although the integration of CO_2_ capture and conversion is very promising to lower the CO_2_ concentration in the atmosphere, there are still many challenges ahead. The amine-based absorption is currently employed by the industry, however, their toxicity, corrosiveness and high cost limit their further market applications. Suitable amine-based or solid amine-based materials with low-energy, low-cost integrated CO_2_ capture and conversion capabilities remain to be developed. For POP-based materials, their long-term and cycling stability, the impact of SO_x_, NO_x_, and other gases remain unclear. The production cost and complexity should be evaluated for future commercial promotion. The electrochemical membrane reactors integrated with catalysts is very promising for high-temperature CO_2_ capture and conversion, but the CO_2_ permeation flux and long-term stability are still needed to be improved. For the DFM-based materials, researching how to achieve the match between CO_2_ capture and conversion is critical. The issues related with catalyst deactivation and operational life also remain to be solved.

To expedite innovation in the chemical industry, an integrated approach combining chemical experimentation with machine learning algorithms can be pursued. This paradigm shift will enable researchers to identify optimal materials faster and more efficiently, ultimately accelerating the pace of innovation in the global chemical industry.

## Figures and Tables

**Table 1:** Cycloaddition of epichlorohydrin with CO_2_ to form cyclic carbonate over various catalysts at 29 °C [21].

Entry	Catalysts	Additives	Time (h)	Conv. (%) ^a^	Select. (%) ^a^
1 ^b^	POP-TPP	n-Bu_4_NBr	24	52.1	>99.0
2 ^c^	Co/POP-TPP	n-Bu_4_NBr	24	95.6	99
3 ^c^	Co/TPP	n-Bu_4_NBr	24	97.5	99
4	Co/POP-TPP	None	24	9.7	99
5	Co/TPP	None	24	18.5	99
6	None	n-Bu_4_NBr	24	34	99
7 ^d^	Co/POP-TPP	n-Bu_4_NBr	96	96.1	99
8 ^e^	Co/POP-TPP	n-Bu_4_NBr	24	88.9	99
9 ^f^	Zn/POP-TPP	n-Bu_4_NBr	24	93.2	>99.0
10 ^f^	Zn/TPP	n-Bu_4_NBr	24	93.5	>99.0
11 ^g^	Mg/POP-TPP	n-Bu_4_NBr	24	80.5	>99.0
12 ^g^	Mg/TPP	n-Bu_4_NBr	24	99.3	>99.0
13 ^h^	Co/POP-TPP	n-Bu_4_NBr	24	93.6	99

Note: ^a^ Determined using GC analysis. ^b^ 48 mg of POP-TPP was used. ^c^ 50 mg of Co/POP-TPP (1.6 mg Co) or 19 mg of Co/TPP (1.6 mg Co) was used. ^d^ Co/POP-TPP catalyst (12.5 mg, 0.4 mg Co). ^e^ 50 mg of n-Bu_4_NBr. ^f^ 50 mg of Zn/POP-TPP (2.3 mg Zn) or 23.5 mg of Zn/TPP (2.3 mg Zn) was used. ^g^ 50 mg of Mg/POP-TPP (1.4 mg Mg) or 37.0 mg of Mg/TPP (1.4 mg Mg) was used. ^h^ Recycled for 18 times.

**Table 3:** Carbon dioxide methanation dual-functional materials summary.

DFM	Condition (°C)		Ref.
	Absorption	Reaction	
Ni-CaO/Al_2_O_3_	280–520 10% CO_2_/Ar	280–520 10% H_2_/Ar	[55]
Ni-Na_2_CO_3_/Al_2_O_3_	280–520 10% CO_2_/Ar	280–520 10% H_2_/Ar	
Ni-Na_2_O/Al_2_O_3_	320 7.5% CO_2_/N_2_ and 7.5% CO_2_, 4.5% O_2_, 15% H_2_O/N_2_	320 15% H_2_/N_2_	[52]
Ru-Na_2_O/γ-Al_2_O_3_	320 15% CO_2_/N_2_	320 20% H_2_/N_2_	[57]
Rh-CaO/γ-Al_2_O_3_	320 10% CO_2_/N_2_	320 2% H_2_/N_2_	
Ru-CaO/γ-Al_2_O_3_	280–400 1.4% CO_2_/Ar and 11% CO_2_/Ar	280–400 10% H_2_/Ar	[54]
Ru-Na_2_CO_3_/γ-Al_2_O_3_	280–400 1.4% CO_2_/Ar and 11% CO_2_/A	280–400 10% H_2_/Ar	
Ru-CaO/γ-Al_2_O_3_	320 10% CO_2_/air and 8% CO_2_/21% H_2_O/air	320 5% H_2_/N_2_	[51]
Ru-CaO/γ-Al_2_O_3_	320 10% CO_2_/N_2_	320 4% H_2_/N_2_	[57]
Ru-CaO/γ-Al_2_O_3_	320 7.5% CO_2_, 4.5% O_2_, 15% H_2_O/N_2_	320 5% H_2_/N_2_	[58]
Ru-Na_2_CO_3_/γ-Al_2_O_3_	320 7.5% CO_2_/N_2_ and 7.5% CO_2_, 4.5% O_2_, 15% H_2_O/N_2_	320 5% H_2_/N_2_	[59]
Ru-Na_2_O/γ-Al_2_O_3_	250–350 7.5% CO_2_, 4.5% O_2_, 15% H_2_O/N_2_	250–350 15% H_2_/N_2_	[60]

## Data Availability

Not applicable.
